# Childhood Adversity and Reactive Attachment Disorder in Adolescents: A Systematic Review

**DOI:** 10.1007/s40653-026-00830-5

**Published:** 2026-02-10

**Authors:** Florencia Talmón-Knuser, Lidia Flores-Cantos, Alba Espuig, Francisco González-Sala, Laura Lacomba-Trejo

**Affiliations:** 1 Department of Developmental Psychology and Education, Faculty of Health Science, Catholic University of Uruguay, Montevideo, 11600 Uruguay; 2 Faculty of Psychology and Speech Therapy, Universitat de València, Valencia, 46010 Spain; 3 Department of Developmental and Educational Psychology, Faculty of Psychology and Speech Therapy, Universitat de València, Valencia, 46010 Spain

**Keywords:** Adverse childhood experiences, Reactive attachment disorder, Adolescents, Systematic review

## Abstract

Adverse Childhood Experiences (ACEs), particularly those related to caregiving, can compromise the attachment bond that children establish with their primary caregivers. This study aimed to understand, through a systematic review, the relationship between ACEs that occurred in early life and the symptoms of Reactive Attachment Disorder (RAD) during adolescence. Using the PRISMA methodology, a search was conducted in the Web of Contents databases Science, ProQuest Central, PubMed and Scopus. The search yielded 1,443 results, of which only 8 met the inclusion criteria. The results show that there is a relationship between ACEs and a greater presence of RAD symptoms, as well as other mental health problems in adolescents. Likewise, those adolescents who are under some protective measure and have experienced ACEs, present more symptoms associated with RAD. It can be concluded that ACEs have negative consequences on establishing secure bonds, leading to more significant development of RAD and its maintenance during adolescence. The results can help to propose prevention and intervention strategies in vulnerable groups.

## Introduction

Adolescence is the life cycle stage from childhood to emerging adulthood, from 10 to 19 years of age. It is a crucial moment in the life cycle for optimal development (World Health Organization [WHO], 2024), in which the quality of the emotional bonds established in childhood, specifically attachment, will be fundamental (Márquez & Gaeta, 2017).

According to Bowlby (1952), attachment is the deep and lasting emotional connection with others, distinguishing which person is trustworthy or not. Interactions with caregivers greatly influence the attachment bond, that is, the responses that the primary caregiver provides to the child’s needs (Lacomba-Trejo, 2022; Pons-Salvador et al., 2009; Zeanah & Gleason, 2015). To grow mentally healthy, the primary caregiver and the young child must experience a warm, intimate, and ongoing relationship that both perceive as satisfying and enjoyable (Bowlby, 1952). Depending on how sensitive these interactions are, the infant will develop a secure, insecure or disorganised attachment (Main & Solomon, 1990). These early experiences, therefore, will serve as the basis for future emotional regulation, behaviour and relationships (Creamer & Hand, 2022).

In this sense, adverse childhood experiences (ACEs) are associated with the development of insecure and disorganized attachments (Carlson, 1998; Cyr et al., 2010; Snyder et al., 2024; Ye et al., 2023), experiencing fear or anxiety when interacting with attachment figures, who should provide security and warmth (Bowlby, 1952). Thus, ACEs are those experiences during childhood and adolescence that are related to abuse, neglect and/or loss (Anda et al., 2010; Goddard, 2021), causing social, emotional and cognitive deterioration; followed by risk behaviors, which could lead to early death (Felitti et al., 1998). There are numerous types of ACEs, including: domestic violence, physical or emotional neglect by caregivers, household dysfunction (mental health problems in the family, gender-based violence, incarceration or substance abuse), physical, sexual or emotional assault or abuse, traumatic injury or illness (Felitti et al., 1998; Felitti, 2009). Recently, due to their negative implications on adult health, other ACEs have been added such as school and community violence, natural disasters, forced displacement, war, terrorism and political violence (Anda et al., 2010; Hughes et al., 2017). In terms of prevalence, it is known that at least 40% of the child and adolescent population will experience four or even more ACEs throughout childhood and adolescence (Bucci et al., 2016).

ACEs are associated with mental health problems, such as depression, risk behaviors, substance abuse, post-traumatic stress disorder (PTSD) or Reactive Attachment Disorder (RAD); as well as with physical health problems (lung and heart disease, sexually transmitted infections or obesity) (Goddard, 2021). In this sense, due to its direct association with ACEs, especially those related to neglect or loss, the RAD is of great interest. RAD is as the difficulty in establishing bonds with others, characterized by limited positive affect or unexplained episodes of irritability. For the diagnosis, it is necessary to have experienced a pattern of grossly insufficient and negligent care, impacting on the establishment of secure and selective attachments (APA, 2022).

RAD can trigger emotional, behavioral, and neurodevelopmental problems, as well as alterations in social ties (Talmón-Knuser et al., 2023), leading to superficial relationships (Hornor, 2019). Adolescents with RAD, compared to other groups, have problems with self-regulation (Seim et al., 2021), and a greater presence of internalizing and externalizing (Kurth et al., 2004) symptoms such as anxiety, depression, anger, and traumatic experiences (Shimada et al., 2015).

For all the above, knowing how ACEs can be linked to the development or suffering of RAD during adolescence is especially relevant. Thus, this research aims to establish the connections between ACEs in childhood and the clinical manifestations of RAD in adolescence. To do so, the following research question is raised, based on the PICO strategy: What is the impact of ACEs (Adverse Childhood Disorders) on the development and maintenance of RAD during adolescence? (Santos et al., 2007).

## Materials and Methods

### Search Strategy

ACEs and RAD were carried out according to the PRISMA standard (Page et al., 2021). The search protocol was previously registered in PROSPERO (ID: CRD42024551113). The search ended in February 2024, and was conducted at: Web of Science, ProQuest Central, PubMed and Scopus. The expression Boolean that was used was the following: TS= (adverse childhood experiences OR child* adversit* OR child* maltreatment OR child* abuse OR family dysfunction OR neglect OR household dysfunction* OR negative childhood experienc* OR child* trauma AND attachment disorder OR reactive attachment disorder AND adolesc*) in the WOS case; noft (adverse childhood experiences OR child* adversit* OR child* maltreatment OR child* abuse OR family dysfunction OR neglect OR household dysfunction* OR negative childhood experienc* OR child* trauma) AND noft (attachment disorder OR reactive attachment disorder) AND noft (adolesc*) at ProQuest; and [Title/Abstract] OR reactive attachment disorder [Title/Abstract]) AND (adolesc* [Title/Abstract]) in the PubMed database; TITLE-ABS-KEY (adverse AND childhood AND experiences OR child* AND adversit* OR child* AND maltreatment OR child* AND abuse OR family AND dysfunction OR neglect OR household AND dysfunction* OR negative AND childhood AND experienc* OR child* AND trauma) AND TITLE-ABS-KEY (attachment AND disorder OR reactive AND attachment AND disorder) AND TITLE-ABS-KEY (adolesc*) in Scopus case.

### Eligibility Criteria

Regarding the inclusion criteria, the following were established: (1) studies that were in high-impact journals from the year of publication of the DSM-5 (2013), a year in which the diagnostic criteria were established differently from the previous edition for attachment disorders, giving rise to two different entities (RAD and Disinhibited Social Engagement Disorder, DSED), (2) in any existing language, (3) in which a study was carried out that evaluated the relationship of ACEs with RAD, in (4) participants between 10 and 19 years old.

The following types of documents were excluded: (1) narrative reviews, single-case designs, narrative or theoretical studies, doctoral theses or grey literature, (2) articles that, if they covered several consequences of ACEs, did not describe in detail the results obtained for RAD, and (3) articles that included only information on DSED.

### Procedure

The articles obtained were uploaded to HubMeta (Hubmeta Systematic Review and Meta Analysys Cloud [Internet], 2020)a program specialized in screening and analyzing online data. The number of articles analyzed was 1,443, of which 362 were automatically discarded for being duplicates. Subsequently, two independent blinded authors (LF-C & LL-T) reviewed each study’s titles and abstracts, and in a second phase, the full texts were reviewed. If there was any disparity in the final decision, a third blinded expert in the field (FG-S) was consulted.

In addition, both authors (LF-C and LL-T) reviewed the reference lists of the selected studies to assess the possibility of including quality references that had not been detected in previous searches. Finally, a manual search was conducted using snowball sampling in order to identify notable articles, and no new records were found within the inclusion criteria.

Subsequently, Cohen’s Kappa (*κ*) was calculated (Orwin & Vevea, 2009) to assess the inter-rater agreement index. Values between − 1 and 0.40 are understood as unsatisfactory, values between 0.41 and 0.75 as satisfactory, and values ≥ 0.76 as satisfactory (Hernández-Nieto, 2002). Due to the wide diversity of results, dependent and independent variables, it was considered that a further meta-analysis of these data would not be appropriate.

### Methodological Quality of the Selected Articles

The quality of the selected studies was assessed by two authors (LF-C & LL-T) in a blinded and independent manner using an adapted version of the “Quality Assessment Tool for Quantitative Studies”, developed by the Effective Public Health Practice Project (Thomas et al., 2004). Seven criteria were considered: study design, representation - selection bias, representation II - dropouts and withdrawals, confounding factors, data collection, data analysis, and results (Table 1).

**Table 1:** Sample characteristics and design of the studies included in the review

FIRST AUTHOR	STUDY DESIGN	REPRESENTATION	REPRESENTATION II	CONFOUNDING FACTORS	DATA COLLECTION	DATA ANALYSIS	RESULTS	TOTAL
KAY	3	1	No follow-up	2	2	1	1	Moderate
SHIMADA	3	4	No follow-up	1	1	3	1	Moderate
MORAN	4	4	No follow-up	2	1	1	1	Moderate
FUJISAWA	5	4	No follow-up	3	2	1	1	Moderate
MARKOTA	5	2	No follow-up	3	3	2	1	Moderate
VIVRETTE	2	3	No follow-up	2	2	1	1	Moderate
LEHMANN	3	1	No follow-up	3	1	1	1	Moderate
SEIM	3	2	No follow-up	3	2	1	1	Moderate

Subsequently, a mean was calculated to classify articles as weak, moderate or strong. This score ranged from 1 to 5, where a score of 1 indicated low risk of bias and thus high methodological quality, while a score of 5 indicated high risk and low methodological quality in the study (Ávila et al., 2016; Glonti et al., 2015; Stroup et al., 2000).

## Results

### Study Selection and Screening

**Fig. 1:**
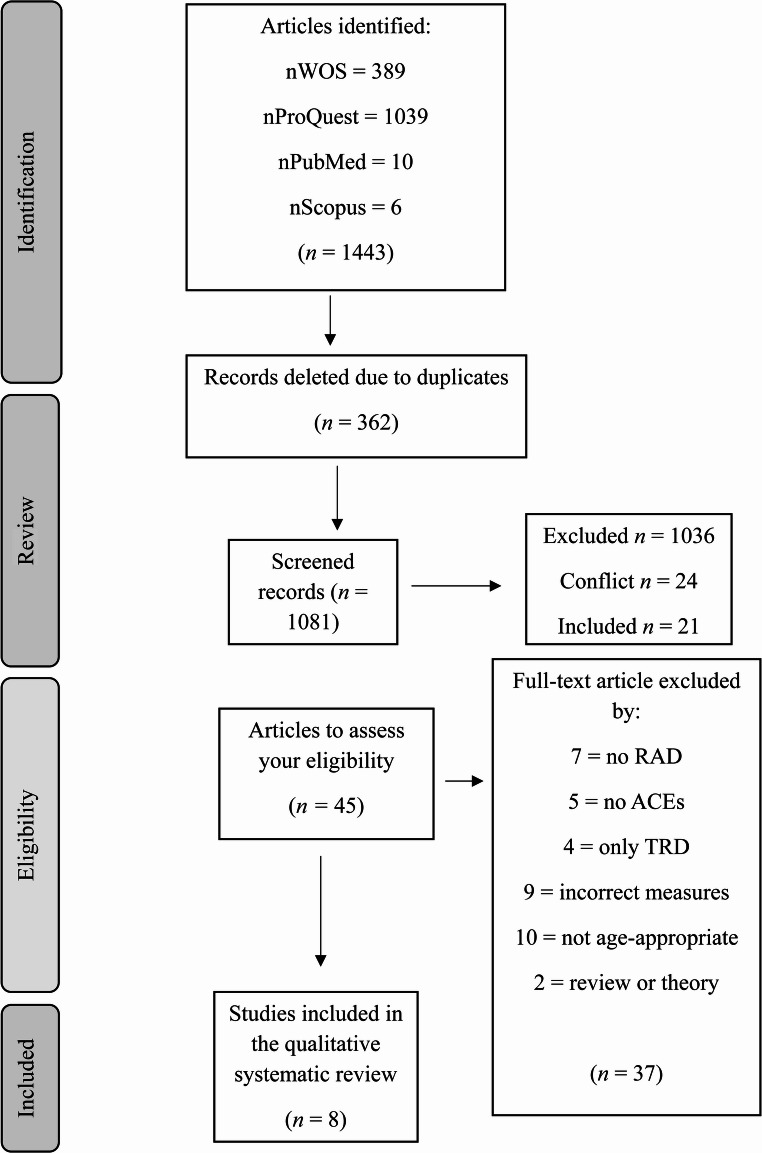
Flow of information through the different phases of a systematic

After importing the information into Hubmeta, and removing duplicate studies (*n* = 362), a total of 1,081 studies were obtained. First, based on the title and abstract of each article, the two authors excluded 1,036 articles for not meeting the previously established inclusion and exclusion criteria. The inter-rater consistency between the independent raters at this stage was high (*κ* = 0.73). Secondly, the full texts of the remaining 45 articles were read. Thus, 37 studies were excluded, and finally 8 studies were eligible for inclusion in the qualitative analysis. In this second screening, the degree of inter-rater agreement was almost perfect (*κ* = 0.95) (Fig. 1).

### Participants and Design

The 8 selected articles included a total of 5,444 adolescents, including 45.1% boys and 54.9% girls. Of these participants, 4,844 (88.9%) experienced some type of ACE (Fujisawa et al., 2018; Kay & Green, 2013; Markota et al., 2018; Moran et al., 2017; Seim et al., 2022; Shimada et al., 2015; Vivrette et al., 2018). Specifically, the most frequently reported ACEs included emotional, physical, and sexual abuse, neglect, exposure to domestic violence, traumatic loss, and community violence (Kay & Green, 2013; Lehmann et al., 2020; Moran et al., 2017; Seim et al., 2022; Shimada et al., 2015).

Regarding their clinical characteristics, 498 (9.15%) had a diagnosis of RAD (Fujisawa et al., 2018; Kay & Green, 2013; Markota et al., 2018; Moran et al., 2017; Seim et al., 2022; Shimada et al., 2015), 3,988 (73.25%) were patients in a Childhood Traumatic Stress (CTS) inpatient unit (Vivrette et al., 2018), and 44 (0.80%) were typically developing control groups with no history of ACEs who were compared to participants with a history of ACEs, those in adoption or foster care, and those diagnosed with attachment or other disorders (Fujisawa et al., 2018; Shimada et al., 2015). Regarding the design, all studies are cross-sectional, of which two of them are retrospective (Fujisawa et al., 2018; Markota et al., 2018). All these data can be found in Table 2..

**Table 2.** Sample characteristics and design of the studies included in the review

Article	Country	Sample	Design
Kay & Green (2013)	UnitedKingdom	153 high-risk adolescents in out-of-home care (LAC) aged 10–16 years (M = 14.34, SD = 1.92), 52% male. The sample had been out of family care for several years following severe early family maltreatment; most commonly emotional abuse, but also sexual abuse. All showed RAD symptom data. 97% had experienced at least 1 category of maltreatment (M = 2.6). 42 low-risk (LR) community controls with RAD diagnosis (M = 14 years, SD = 1.5), 38% men, with no history of abuse by caregivers. 121 social workers and 145 caregivers.	Nonexperimental, transversal, comparative
Shimada et al. (2015)	Japan	21 adolescents aged 10–17 years (M = 12.76) with a clinical diagnosis of RAD who hadexperienced physical abuse (n = 7), emotional abuse (n = 11), sexual abuse (n = 2), and neglect(n = 15) early in life and were currently living in a child welfare facility; and 22 typicallydeveloping adolescents (TDA) with no history of maltreatment (M = 12.95) recruited from localschools.	Nonexperimental, exploratory, comparative
Moran et al. (2017)	UnitedKingdom	29 adolescents from Intensive Services, aged 12-17 years (M = 16.2, SD = 1.3), 10 girls and 19 boys. 86% had experienced some form of maltreatment and the rate of actual or borderline RAD was 52% (15 participants). 19 had experienced emotional neglect and 11 physical, 10 emotional, 12 physical and 7 sexual abuse and 18 witnessed domestic violence. 29 residential care staff carers and 20 teachers to report.	Transversal, nonexperimental, correlational
Fujisawa et al. (2018)	Japan	21 adolescents aged 10–17 years with a clinical diagnosis of RAD (M = 12.8 years; 13 girls) were recruited from the Department of Child and Adolescent Psychological Medicine, Fukui University Hospital. All children had experienced physical and/or emotional abuse and/or neglect at an early stage. 7 (33.3%) of the participants had experienced physical abuse, 11 (52.4%) emotional abuse, 2 (9.5%) sexual abuse, and 16 (76.2%) neglect. The mean duration of exposure was 7.7 ± 4.68 years. 22 typically developing adolescents (TDA) with no history of maltreatment as control participants from schools or local communities (M = 12.95 years; 12 girls).	Nonexperimental, transversal, retrospective, comparative
Markota et al. (2018)	USA	Sample of 366 adolescents (71% female) aged 11-18 years (M = 14.9, SD = 1.8) with RAD and ACEs diagnoses who had been hospitalized several times in the psychiatric unit during 2015. Medical records were used to confirm that patients were victims of ongoing bullying at the time of first hospitalization (no differentiation was made between types of bullying).	Retrospective nonexperimental, correlational design
Vivrette et al. (2018)	USA	3988 cases between 7 and 18 years of age (M = 10.61) collected from the National Childhood Traumatic Stress Network (NCTSN) Core Data Set (CDS). All had experienced some type of ACE, and 1328 participants had experienced child care deficiencies. It included an assessment of exposure to 19 specific types of traumatic events, including abuse, neglect, domestic violence, natural disasters, community violence, and school violence, as well as poor caregiving (requiring the primary caregiver to be unable to: provide adequate care, guidance, and support to the child; and attend to the child's basic developmental needs). Groups were formed based on characteristics of caregivers' caregiving deficiencies (drug problems, mental health problems, etc.) Some tests were completed by caregivers and physicians.	Nonexperimental, comparative, transversal
Lehmann et al. (2020)	Norway, Canada, United Kingdom	The total sample (N =399, 53.9% response rate) consisted of caregivers such as foster parents (n =320), 277 foster mothers and 43 foster fathers; or foster youth (n =302). They were between 11 and 17 years old (M =14.8, SD =2.05), 53% were male.	Nonexperimental, crosssectional, correlational
Seim et al. (2022)	Norway	381 participants aged 12.2–20.2 years (M = 16.7, SD = 1.4) with data on rad and DSED who had lived in RYC in Norway. 57.7% were girls, and 78.2% were of Norwegian ethnicity. 71% reported exposure to maltreatment. We previously reported that the range of symptom frequencies for RAD and DSED was 2–35 and 4–11%, respectively, and the prevalence rates of diagnosis were 9% for RAD (n =33) and 8% for DSED (n =31). Primary contacts of participants were also contacted.	Nonexperimental, crosssectional, descriptive

*Note*. LAC – Look After Care; LR - Low-risk; DTI - Typical development; OR - Odds ratio; CI - Confidence interval; M – Mean; SD – Standard deviation; TA – Attachment disorder; RAD (Reactive Attachment Disorder); DSED (Disinhibited Social Engagement Disorder); MG – Gray matter

### RAD Symptoms When Suffering from ACEs

Adolescents in care had more RAD symptoms than the low-risk group. Participants entering care at a younger age had more RAD symptoms and higher scores on superficial relationships (Kay & Green, 2013). ACEs, specifically physical and psychological violence by caregivers, neglect, and sexual abuse, were directly associated with RAD, specifically low social-emotional ability and emotional dysregulation (Lehmann et al., 2020). Children exposed to maltreatment between the ages of 4 and 7 years showed higher scores on anxiety, PTSD, and dissociation symptoms in the RAD group than typically developing children (Fujisawa et al., 2018).

### Relationship of RAD with Other Difficulties

Adolescents with RAD exhibited higher levels of both internalizing and externalizing problems compared to peers without RAD. Elevated rates of attention-deficit/hyperactivity disorder (ADHD), autism spectrum disorder (ASD), depression, anxiety, post-traumatic stress symptoms, and dissociation were reported across studies (Moran et al., 2017; Shimada et al., 2015). RAD symptoms were also associated with hyperactivity, peer-related difficulties, and poorer adaptive functioning across multiple domains. Moreover, increasing RAD symptom severity was linked to a greater likelihood of comorbid mental health conditions, self-harm behaviors, and suicidal ideation. RAD were highly associated with increased symptoms on all subscales assessing behavior, as well as poorer (lower) adaptive functioning scores and greater impairments in multiple domains of social and physical functioning (*p* <.01) (Kay & Green, 2013).

### Psychopathology and Neuropsychological Outcomes Related to ACEs

The younger the children entered care, the more superficial their relationships were (Kay & Green, 2013). Adolescents diagnosed with RAD and who had experienced ACEs were more likely to be hospitalized in psychiatric units (Markota et al., 2018). Poor caregiving quality and caregiver-related difficulties, such as substance use and mental health problems, were linked to higher levels of internalizing behavior, post-traumatic stress symptoms, and RAD symptomatology (Vivrette et al., 2018).

Of foster youth, 80% reported experiencing ACEs. Of this sample, PTSD symptoms were higher in girls and lower in adolescents seen in mental health clinics. Most potentially traumatic events were associated with PTSD. Exposure to maltreatment between ages 4 and 7 years, ACEs, and the number of types of maltreatment, as well as neglect, were significant predictors of gray matter variations (Fujisawa et al., 2018). Participants with RAD showed reduced gray matter volume in the left primary visual cortex and left inferior occipitotemporal cortex compared to the typically developing group (Fujisawa et al., 2018; Shimada et al., 2015; Lehmann et al., 2020) (Table 3).

**Table 3:** Studies included in the review: main findings and conclusions

Article	Variables	Main Results	Conclusions
Kay & Green (2013)	Demographic variables. History of abuse and care (questionnaires administered by social workers). Psychological variables: RAD, social and physical functioning, and functioning adaptive	The LAC group (*M *= 13.4, *SD *= 8) showed significantly higher total scores than the LR group (*M *= 3.5, *SD *= 3) on RAD. 63% (96/153) of LAC adolescents met criteria for total RAD symptoms (≥9.8); 58% (89/153) for TRD (≥2.4); 52% (80/153) for Care Seeking (≥3.5); 56% (86/153) for Superficial Relationships (≥2.8); and 20% (31/153) for Unpredictability (≥3.1). The DAWBA-RAD attention-seeking scale showed an interaction between group, age, and gender, with younger LAC men showing higher attention-seeking scores (*B *= -0.03, *SE *= 0.02, *p *= 0.02; 95% CI [-0.06, -0.01]. Younger age at first entry to care was associated with higher subsequent total RAD scores (*R² *= 0.24; *B *= −0.36; *SE *= 0.18; *p *= 0.05) and higher superficial relationship scores (R² = 0.21; *B *= −0.17; *SE = *0.07; *p *= 0.01). Multiple maltreatment was associated with significantly higher care-seeking scores (*R² *= 0.28; *B *= 0.52; *SE *= 0.24, *p = *0.03).	Early withdrawal from care indicates greater prior risk and early adversity and evidence is found of a relationship between severity of maltreatment and a RAD factor. Earlier entry into care will reduce the overall duration of developmental exposure to maltreatment. A significant proportion of adolescents in care show substantial levels of RAD. These behaviors are associated with severe functional impairment in various aspects of adolescents' lives.
Shimada et al. (2015)	Demographic variables. Clinical and psychological variables: Gray matter volume, type of abuse, depression, post-traumatic symptoms, ADHD and ASD.	Compared with the DTA group, the RAD group showed higher levels of psychiatric symptoms: depressive symptoms (*p *< .01); post-traumatic symptoms and other relevant symptoms, such as anxiety (*p *< .05); depression (*p *< .01); anger (*p *< .01); post-traumatic stress (*p *< .01); and dissociation (*p *< .01). Similar differences (SD < RAD) were found on measures of internalizing behavior (*p *< .001) and externalizing problems (*p *< .01), as well as on tests for ADHD (*p *< .01) and autism traits (*p *< .05); attention shifting *p *< .001); communication (*p *< .01). The RAD group, compared with the DTA group, showed reduced gray matter volume in the left primary visual cortex (*p *= .038). An average 20.6% reduction in gray matter volume was found in the identified regions of the RAD group compared with the DT group. Internalizing behavioral problems (*β *= −.96, *t *= -3.86, *p *< .05) were a significant predictor for left visual cortex gray matter volume estimates in the RAD group, explaining approximately 55% of the variance in gray matter volume (*R2 *= .55) (*F *[10,8] = 3.16, *p *< .10).	Structural abnormalities in the left occipital visual cortex were associated with the presence of RAD. Furthermore, RAD internalization problems were correlated with gray matter volume in the visual cortex. Early adverse experiences may affect the development of the primary visual system, which is reflected in the size of the visual cortex in children and adolescents with RAD. These GMV abnormalities of the visual cortex may also be associated with deficits in visual emotion regulation in RAD.
Moran et al. (2017)	Demographic variables. History of abuse, number of displacements. Clinical and psychological variables: neglect and abuse, child psychiatric symptoms, RAD.	Strong positive correlation between RAD scores and mental health problems (*rs *= 0.60), hyperactivity (*rs *= 0.50) and peer relationship problems (*rs *= 0.47). In the instruments answered by the teachers, there was a strong correlation was found between the total scores of the TA and the conduct (*rs *= 0.54) The only correlation that was significant was that between TA total scores and hyperactivity. There is a higher percentage of people with AD who have possible other mental health problems, emotional difficulties (60% vs. 36%), behavioral problems (100% vs. 71%), hyperactivity (67% vs. 21%), and problems with peers (87% vs. 71%).	There was a high level of history of abuse in the participants. High rates of attachment disorders are observed among young people who have committed high-risk offences and who receive specialist services. Attachment disorders are strongly related to other mental health problems in this population.
Fujisawa et al. (2018)	Demographic variables. Clinical and psychological variables: abuse, and neglect, laterality, brain imaging, anger, depression, post-traumatic stress, dissociation.	Volume was significantly reduced by 20.6% in the left primary visual cortex of the RAD group compared with the DTA group (*BA 17; *MNI coordinates, *x* = -20, *y *= -74, *z *= 8; cluster size = 644 voxels, *p *= 0.038). Exposure to maltreatment between the ages of 4 and 7 years was the most significant predictor of MG variables in the left primary visual cortex of the RAD group and in anxiety, PTSD, and dissociation problems (*p* < .01). In degree of GM variations in the left visual cortex of the RAD group the number of types of maltreatment was the most important predictor (*p* < 0.05). Neglect was the second most important predictor (*p* < 0.05).	The type and timing of maltreatment play an important role in inducing structural abnormalities in the left occipital visual cortex in children with RAD. The results suggest that ACEs may affect the development of the primary visual system, reflected by reduced MG volume in the visual cortex.
Markota et al. (2018)	Demographic variables: Clinical and psychological variables: hospitalisation history, RAD, PTSD (from Advanced Cohort Explorer (ACE).	In adolescents with trauma, the effect of trauma remained significantly associated with an increased risk of rehospitalisation (*OR *= 3.2, *CI *= 1.8–5.6, *p *< .0001). Bullied adolescents remained significantly associated with increased risk of rehospitalization (*OR *= 2.2, *CI *= 1.2–3.9, *p *= .009). Youth with a history of trauma were significantly more likely to experience ongoing bullying (*OR *=1.9, *CI *=1.2–3.2, *p *= .01). Trauma (*OR *= 3.0, *CI *=1.7–5.3, *p *< .001) and bullying (*OR *=1.9, *CI *=1.1–3.5, *p *=0.03) remained significantly associated with rehospitalization. History of trauma (odds ratio (*OR*) = 3.2, 95% confidence interval (*CI*) = 1.8–5.6, *p* < .001) and ongoing bullying (*OR *= 2.2, *CI *= 1.2–3.9, *p *= .009) were significantly associated with increased rates of rehospitalization.	History of ACEs and ongoing bullying, commonly found in patients with RAD and PTSD, are risk factors for psychiatric rehospitalization in children and adolescents. Since the highest risk of readmission occurs within the first month after discharge, the findings underscore the need for close and comprehensive follow-up after hospitalization.
Vivrette et al. (2018)	Demographic variables. Clinical and psychological variables: Impairments in Caregiving, Trauma History and behavior problems.	Poor caregiving was significantly associated with confirmed exposure to all forms of intrafamilial trauma (*p *< .001): sexual abuse, physical abuse, emotional abuse, neglect, and domestic violence, as well as traumatic loss/separation/grief. It was also significantly associated with exposure to extrafamilial trauma (*p *< .001): physical assault, interpersonal violence, and community violence. Poor caregiving was significantly related to PTSD symptom severity score consistent with increasing severity in the poor caregiving groups; the “no poor caregiving” group had the lowest mean total score (*M *= 26.4; *SD *= 14.8), whereas the “alcohol/drug problems, mental health problems, and poor caregiving” group had the highest mean total score (*M *= 29.1; *SD *= 14.7). Poor caregiving was also significantly related to scores on the total behavior problems, mainly with *the “mental health problems and poor caregiving” and “alcohol or drug, mental health, and poor caregiving” groups (M = 65.9; SD *= 9.5 and M = 66.1; SD = 9.2), and internalising problems (*M *= 65.9; *SD *= 9.5 and *M *= 66.1; *SD *= 9.2). It is these groups who are significantly more likely to report attachment and behavioural problems.	Exposure to neglectful care is common in trauma-exposed youth. Lack of routine identification of mental illness in caregivers may contribute to higher rates of alcohol/drug-related problems than mental health problems. Youth with caregivers with mental health issues face higher rates of trauma exposure and psychosocial problems, underscoring the importance of proactively addressing caregiver mental health. ACEs are directly related to RAD and other mental health difficulties.
Lehmann et al. (2020)	Demographic variables: age, years lived in foster care, gender, exposure to possible traumatic events (PTE) Psychological variables: RAD and DSED, PTSD.	Adolescents reported experiencing, on average, 3.44 PTEs each (range 0 to 15, SD = 3.33), and 52.9% reported PTSD symptoms at or above the clinical cutoff. Girls reported significantly more PTSD symptoms (*M *= 16.96, *SD *= 15.63) than boys (*M *= 8.93, *SD *= 10.63, *t *= -5.55, *df *= 297, *p *< .001). Of the 18 PTEs, 16 were associated with the PTSD factor. The association between PTSD and four PTEs yielded large effect sizes (*r *≥ .5). Nine of the PTEs were associated with RAD B. The total PTE score was associated with the latent factors of PTSD (*r *= .66, *p *< .001), RAD B (Low socioemotional responsiveness/emotional dysregulation *r *= .28, *p *< .001), and DSED (*r *= .11, *p *= .046), but not with RAD A (Failure to seek/accept reassurance). The TRD dimension was associated with TAR B (*r *= .31, *p *< .001) and DSED (*r*= .19, *p *< .028).	This study highlights the high prevalence of potentially traumatic events among youth in foster care, as well as the significant association between these events and symptoms of PTSD and attachment disorders. There is overlap between PTSD and ACE symptoms, but they represent distinct manifestations of trauma sequelae.
Seim et al. (2022)	Demographic variables: Psychological variables: psychiatric disorders, RAD and DSED, ADHD, emotional and behavioral problems.	Among adolescents with a diagnosis of RAD, all disorders were prevalent, with 65% meeting criteria for at least one additional psychiatric disorder. All categorical psychosocial problems were more prevalent among adolescents with an RAD diagnosis, with 92% reporting at least one co-occurring psychosocial problem. Adolescents with an RAD diagnosis more RAD symptom burden had clinically significant regression coefficients and statistically significant associations with all CBCL syndrome scales except challenging behavior (*p *= .48). All disorders were prevalent among adolescents with a DSED diagnosis, with 90% meeting criteria for at least one additional psychiatric disorder. The odds of ADHD were 2.5 times greater, and the odds of any other psychiatric disorder 3.5 times greater, for adolescents with a RAD diagnosis than for those without. Adolescents with a RAD diagnosis had a mean of 1.92 comorbid disorders (*p* = .006) and a mean of 4.04 concurrent psychosocial problems (*p* = .006) higher than adolescents without DSED).	RAD and DSED are often misdiagnosed in children and adolescents with histories of neglect. Most adolescents with RAD and DSED also have other psychiatric disorders and psychosocial problems. Both emotional and behavioral problems were found to co-occur with RAD, while DSED was mainly associated with ADHD and emotional problems such as depression and anxiety.

*LAC* Look After Care, *LR* Low-risk, *DTI* Typical development, *OR* Odds ratio, *CI* Confidence interval, *M* Mean, *SD* Standard deviation, *TA* Attachment disorder, *RAD* (Reactive Attachment Disorder), *DSED* (Disinhibited Social Engagement Disorder), *MG* Gray matter

## Discussion

In accordance with the stated objective, which was to determine the impact of ACEs on the development and maintenance of RAD in adolescents through a systematic review, it can be concluded that the included studies consistently associate ACEs with the development or maintenance of RAD. The results indicate that, during adolescence, a history of ACEs is related to an increased presence and severity of RAD symptoms, as well as to higher levels of mental health and neurodevelopmental difficulties compared to typically developing peers (Fujisawa et al., 2018; Kay & Green, 2013; Lehmann et al., 2020; Markota et al., 2018; Moran et al., 2017; Seim et al., 2022; Shimada et al., 2015; Vivrette et al., 2018). It was also observed that the number of ACEs affects brain development, IQ and grey matter (Fujisawa et al., 2018), with a decrease in grey matter volume in the left primary visual cortex and in the left inferior occipitotemporal cortex compared to those without RAD (Shimada et al., 2015). In turn, they presented psychosocial difficulties in their relationships with their peers and at behavioral levels (Kay & Green, 2013; Moran et al., 2017; Seim et al., 2022).

These data are in line with previous literature, which highlights the great impact of experiencing ACEs (Campbell et al., 2016;Felitti et al., 2009); Felitti et al., 1998; Snyder et al., 2024; Ye et al., 2023) and with those who pointed out that people with RAD have a high comorbidity with other mental health and neurodevelopmental problems (Hong et al., 2017; Kurth et al., 2004; Talmón-Knuser et al., 2023, 2024).

However, they highlight in this association the relevance of experiencing ACEs during early ages of development, affecting the establishment of secure bonds between the minor and his/her primary caregiver. Neglect of the care received, continuous changes of caregivers, lack of communication and affection received by adults, affect the development of attachment bonds, leading to the establishment of insecure or disorganized attachments, being more prone to present symptoms of RAD; developing psychosocial, emotional and behavioral problems in later stages (Lehmann et al., 2020; Markota et al., 2018; Seim et al., 2022).

The work points out how those adolescents who experienced ACEs, especially those associated with negligent care and domestic violence, showed more RAD, DSED, PTSD and behavioral problems (Vivrette et al., 2018). This highlights the need to develop awareness and sensitization programs aimed at families, in which issues related to attachment, skills to form secure attachments, emotional regulation and parental reflective capacity are addressed so that caregivers can provide more sensitive and appropriate responses to the specific needs of their children (Císcar et al., 2021).

Evidence also suggests that earlier exposure to ACEs is associated with greater severity of RAD symptoms and increased vulnerability to psychopathology, including PTSD, anxiety, and dissociation. Moreover, earlier and more severe exposure to adversity has been linked to neurodevelopmental alterations, such as reductions in gray matter volume, which may contribute to the persistence of these difficulties over time (Fujisawa et al., 2018; Kay & Green, 2013). Thus, a recent systematic review has shown that withdrawn maternal behaviors may contribute to the development of insecure or disorganized attachment patterns. However, ACEs —particularly severe neglect or deprivation—may lead to the development of RAD. Specifically, evidence suggests that ACEs have direct neurobiological consequences in children with RAD, with alterations observed in brain regions and neural pathways involved in sensory processing and reward systems that mediate emotional regulation, compared to controls without mental health difficulties. Furthermore, the brain of children exposed to severe adversity appears to be less responsive to experiences of reward and achievement (Tomoda et al., 2025).

In addition, ACEs are typically linked to adverse events within the home environment, such as maltreatment, neglect, and exposure to intimate partner violence. These adverse experiences do not necessarily have to be experienced directly. In this sense, exposure to community violence in a direct or vicarious way has been related to the presence of PTSD, especially in those who presented a preoccupied attachment style (Lindberg, 1997). Along the same lines, Thorton (2014), in a study with children who were in homes where there was domestic violence, points out that one factor of post-traumatic stress is violence that is learned indirectly. Howard (2021) points out the vulnerability of children and adolescents to experience victim traumatisation, identifying with the victim (DeVoe & Smith, 2002; Thornton, 2014), an aspect that can be related to empathic responses to the distress experienced by another person, which can manifest itself even in children as young as one year old (Geangu et al., 2010; Hoffman, 2001; Roth-Hanania et al., 2011). In the case of domestic violence, direct but also indirect exposure generates environments characterised by insecurity and a lack of predictability, which can affect how caregivers respond to children’s needs and thus how children bond.

Finally, and related to the above, adolescents who have lived in residential homes or who are in adoption deserve special attention, as they show significantly higher scores for RAD, DSED, and other difficulties compared to those adolescents who have not experienced ACEs (Kay & Green, 2013). This would indicate that children in residential homes are more likely to develop attachment problems and psychological stress, as previously reported in the literature (Schröder et al., 2017; Talmón-Knuser et al., 2023). This suggests an urgent need for preventive interventions and specialized treatment, offering easy access to psychological and psychotherapeutic treatment services for children and adolescents and their foster or adoptive families.

### Limitations

Despite the various strengths of this systematic review, several limitations must be considered when interpreting its results. First, the main limitation of this review is the small number of studies included (*N* = 8), which reflects the limited availability of empirical research on RAD in adolescence and restricts the generalizability of the findings. Second, the small sample sizes in some studies hinder the generalization of the findings to a broader population. Future research should employ probabilistic sampling methods to better represent the characteristics of the adolescent population of interest.

On the other hand, the heterogeneity of the measures used across studies prevented the possibility of conducting a meta-analysis, which would have been desirable to further consolidate the findings. Beyond limiting quantitative synthesis, this heterogeneity also complicates direct comparison across studies and may influence the consistency and magnitude of the reported associations, as similar constructs were operationalized using different assessment tools and outcome measures. Additionally, the strict inclusion criteria may have led to the exclusion of relevant articles, potentially limiting the scope of the review. It should be noted that the findings of this review must be interpreted considering the methodological characteristics of the included studies. Given their predominantly descriptive and observational nature, a formal risk of bias assessment was not conducted. Nevertheless, several study characteristics may represent potential sources of bias, including reliance on caregiver reports, the absence of adolescent self-reports, and limited reporting of informant characteristics. These factors may affect the reliability and validity of the synthesized evidence and should be considered when interpreting the observed associations between adverse childhood experiences and reactive attachment disorder.

Most studies used cross-sectional and retrospective designs, precluding the establishment of causal relationships between ACEs and RAD. As a result, the directionality and temporal sequencing of these associations cannot be determined, and conclusions regarding causality must be interpreted with caution. However, several of the instruments used have shown good reliability (e.g., Pinto et al., 2014). As for the retrospective design, they could be underestimated or overestimated, due to alterations in the participants memories; either due to forgetfulness or incorrect reporting (Gomes et al., 2019). For these reasons, future research should adopt longitudinal designs to explore factors related to the prevention, consequences, and treatment of these issues in at-risk adolescents and their caregivers.

Furthermore, some studies relied solely on caregiver reports without including adolescent self-reports or observational measures, potentially omitting valid data. Only three studies, Kay and Green (2013), Moran et al. (2017) and Lehmann et al. (2020), specified the number of caregivers who participated, and none described key characteristics of the informants, such as gender, age, or mental health history. This lack of information may introduce reporting bias and uncontrolled confounding variables, thereby affect the reliability and validity of the synthesized evidence and contribute to the moderate quality assessment of the analyzed articles.

However, this systematic review has several notable strengths. Firstly, the instruments and statistical methods employed are robust, facilitating consistent and reliable analysis of the results. Despite the difficulty of collecting data in samples that have experienced ACEs, such as institutionalized or hospital settings, the samples presented, although small and non-random, are sufficient. Additionally, most studies utilized data provided by individuals close to the participants, such as primary caregivers, social workers, teachers, and physicians. This approach reduces common self-report errors and provides a more comprehensive understanding of the variables studied.

To our knowledge, this research is the first systematic review to be conducted on the impact of ACEs on RAD development. This review, the result of an exhaustive search of the literature using precise inclusion criteria, expands knowledge beyond the conclusions of previous narrative reviews. In addition, this review had two evaluators who were blinded throughout the process, including the agreement rate between them.

Finally, although this review followed a rigorous and transparent methodology, the exclusion of grey literature and non–peer-reviewed sources may have increased the risk of publication bias, potentially overrepresenting studies with statistically significant findings. Future reviews should consider extending searches to additional data sources, including conference proceedings, book chapters, and doctoral theses, to provide a more comprehensive representation of the available evidence.

### Practical and Theoretical Implications

 The results of this work also suggest important theoretical and practical implications. First, this study allows us to better understand how ACEs influence the development of symptoms of RAD. From the evolutionary–ethological theory of attachment (Bowlby, 1952), a warm, intimate, and continuous relationship between the primary caregiver and the young child is considered essential for healthy psychological development. When this relationship is disrupted or characterized by neglect, deprivation, or chronic conflict, there is a greater likelihood of developing insecure or disorganized attachment patterns (Hornor, 2019).

In this context, emerging evidence suggests that severe ACEs may not only affect attachment relationships but also disrupt neurobiological systems involved in emotional regulation and reward processing, which may help explain the persistence and severity of RAD symptoms. These neurobiological alterations highlight the need for trauma- and attachment-informed interventions that target emotional regulation and relational safety, and they underscore the importance of future longitudinal and intervention studies to examine whether such neural patterns are modifiable over time.

Therefore, mental health professionals must consider the influence of ACEs on both relational and neurodevelopmental processes when assessing and treating adolescents with RAD, emphasizing trauma- and attachment-informed approaches that actively involve caregivers and promote emotional co-regulation. At the policy level, prevention and intervention programs should prioritize early identification of ACEs and the provision of stable, supportive caregiving environments, particularly for children in foster care, adoption, or residential settings.

From a research perspective, future studies should adopt longitudinal designs to clarify developmental trajectories and causal pathways, employ standardized and validated assessment tools to improve comparability across studies, and incorporate multi-informant approaches that include caregiver, adolescent, and observational data to strengthen the reliability and validity of findings.

## Conclusion

In conclusion, the findings of this systematic review indicate that ACEs are associated with a higher prevalence and greater severity of RAD during adolescence, as well as with increased comorbidity with other mental health and neurodevelopmental difficulties.

Early adverse experiences appear to have a significant impact on the development of attachment relationships between the child and the primary caregiver, with consequences for emotional regulation, social functioning, and broader developmental domains (Talmón-Knuser et al., 2023). These disruptions may be further reinforced by alterations in neurobiological systems involved in stress regulation and reward processing, contributing to the persistence and complexity of RAD symptoms over time (Tomoda et al., 2025).

Taken together, these findings highlight the importance of implementing preventive and therapeutic interventions for populations at risk of experiencing ACEs, as well as for groups who have already been exposed to severe adversity, such as children in residential care or adoption contexts. Interventions that promote safe and stable caregiving environments, enhance caregiver mental health and sensitivity, and support emotional co-regulation may help target the underlying mechanisms linking ACEs to RAD, thereby fostering more secure attachment relationships and improving developmental outcomes for children and adolescents.

## Data Availability

Not applicable. No new data were generated or analysed in this study.
